# Sources of Contradictions in the Evaluation of Population Genetic Consequences after the Chernobyl Disaster

**Published:** 2013

**Authors:** V.I. Glazko, T.T. Glazko

**Affiliations:** Russian State Agrarian University – Moscow Timiryazev Agricultural Academy, Timirjazevskaja Str., 49, Moscow, Russia, 127550

**Keywords:** ionizing radiation, molecular genetic markers, reproductive ”success”, cytogenetic anomalies, ecological stress

## Abstract

The review covers the analysis of our own and published data pertaining to population and genetic consequences in various mammalian species under conditions of high levels of ionizing radiation as a result of the Chernobyl accident. The findings indicate that these conditions have promoted the reproduction of heterozygotes in polyloci spectra of molecular genetic markers and animals with a relatively increased stability of the chromosomal apparatus. The prospects of using the reproductive “success” of the carriers of these characteristics as an integral indicator of the selective influence of environmental stress factors are discussed.

## INTRODUCTION

It has been 26 years since the largest technogenic catastrophe of the 20th century – the Chernobyl disaster – which highlighted many of the global issues facing industrial and post-industrial societies. From the point of view of a systemic-evolutionary approach, the processes of anthropogenesis and sociogenesis are the results of employing unique strategies of survival pertaining exclusively to *Homo sapiens*, whose core elements include the simultaneous combination of biological, socio-cultural and technological adaptations. The Chernobyl accident revealed the incompatibility of these elements. Technological adaptation being the most mobile of the listed adaptations, it determines the general direction of the history of humankind. Domestication of plants and animals as a condition of the transition to sedentary life characterized the first phase of the formation of the agrarian civilization and, hence, the creation of novel technologies capable of adapting to dynamic environmental factors. Culture itself (including the production culture) must play a major role in the coordination of all three components of the adaptive strategy. The Chernobyl accident and the attitude towards its consequences and the consequences of numerous technogenic accidents and disasters of the 20th century indicate that culture is developing significantly slower than technological process and its biospheric consequences. The persistent controversies regarding the evaluation of the consequences for the health of the human population not only after the Chernobyl disaster, but also those of the atomic bombings of the cities of Hiroshima and Nagasaki serve as an illustrative example of the latter fact.

Thus far, the consequences of ecological changes for living objects are merely declarative: species on the brink of extinction are counted, their reproductive function is assessed, and changes in communities are evaluated. The inconsistency in the evaluation of the consequences of the Chernobyl accident for biota yet again brings forward an even more global problem; that is a lack of relatively reliable and consistent evaluations of biological safety in human habitats. The urgent need to develop novel approaches for the assessment of biological safety has two reasons: the abrupt increase in the spread of contaminants and the complexity of their composition. The conventional methods for the detection of toxic agents in the air, ground and water do not take into account the constantly emerging novel contaminants and neglect their combined effects, thus requiring an additional analysis of the complex of living organisms which act as a target for toxic agents. It is evident that the selection of indicator species in which population genetic changes can be used as an objective indicator of the biological safety of the region under surveillance remains among the key issues today.

However, the genetic consequences of genotoxic effects are most commonly considered exclusively from the point of view of the risk of emergence of mutant organisms, i.e., the carriers of constitutional mutations that are present in all the cells of a multicell organism. The frequency of occurrence (especially for carriers of such large-scale genetic defects as cytogenetic anomalies) can be used as an indirect measure of the “sensitive” part of the gene pool which does not participate in the reproduction of the population anymore, since the carriers of constitutive mutations in the populations of higher organisms are typically less fertile than the normal individuals.

The main reasons complicating an objective assessment of the damaging effects of ionizing radiation include the following ones: heterogeneity in the radiosensitivity of the investigated groups of organisms at the level of species, populations, individual organisms, various tissues and organs; volatility of the radiosensitivity of the cells isolated from the same organism during the ontogenesis and under the influence of environmental factors (in particular those increasing or suppressing the activity of various parts of the antioxidant system; the complexity of the mutational spectra and their contribution to somatic pathologies, their connection with the reproductive function abnormalities in the organisms.

In order to assess the genotoxicity of a particular factor or the level of regional contamination, the incidence of gene mutation and the number of chromosomal aberrations are typically determined, along with performing a micronuclear test in the members of various indicator species. Thus, for instance, the cytogenetic characteristics of the bone marrow of small mammals are widely used as a biological test to assess the ecological situation in various regions [1–5]. However, the accumulated data clearly indicate that a widespread individual variability exists with respect to the spontaneous frequencies of cytogenetic anomalies and their alterations in response to the genotoxic impact.

The stability of the chromosomal apparatus is a polygenic trait controlled by a large number of various factors and genes, which are not limited to DNA repair enzymes [6]. Thus, aneuploidy is closely connected with the gene mutations that control the synchronism of centrosome division and stages of mitosis. Synchronism impairment results in multicentric mitosis with subsequent errors in chromosome segregation into daughter cells [7–10]. Chronic or transient abnormalities in the telomeric function, mutations or dysfunctions of the genes encoding telomeric proteins lead to their fusion, which causes significant genomic instability [11, 12]. A large number of various sources and mechanisms of genomic instability have been identified at the level of nucleotide sequences; their only common trait is the formation of double-strand breaks (DSB) [13]. It should be emphasized that the frequency of the mutations in various genome segments depends on a number of genomic parameters (nucleotide context, replication duration, nucleosome density, histone modifications, chromatin packing, etc.) [14]. This specific feature typically complicates the assessment of genotoxic effects in genetically heterogeneous populations, including human populations.

The problem is further complicated by the fact that the “release” of mutations in somatic cells, the number of mutants in the progeny, is the final phase of a multiphase process. The observed effect of any genotoxic impact depends on several parameters, including 1) the individual sensitivity of a given organism to the factor under investigation (in particular, the activity of the enzymes participating in the biotransformation of xenobiotics and in antioxidant protection systems), 2) the genotypical characteristics of the activity of the enzymes participating in the repair of the induced defects, 3) the enzymes facilitating the detoxification of the toxic agents that enter the cells and are formed during the intracellular metabolism, and 4) the rate of removal of the damaged cells.

The polygenic nature of the listed functions presumably leads to the widespread individual variability in mammalian groups in response to genotoxic impacts of identical intensity.

It should be emphasized that the underdeveloped conceptions related to the multiplicity of targets for the damaging effect of ionizing radiation in the cytoplasm and nucleus, in various DNA segments and to the no less complex ways of formation of these damages in the form of mutations, and to the protection systems of a multicellular organism, which prevent the accumulation of mutant cells, results in the fact that the predicted damaging effects (for humans in particular) are drastically different from empirically collected data. ;

Taking into account the lack of information regarding the cascades of mutagenesis-related molecular events, the problematic nature of predicting the consequences of an ionizing radiation impact becomes apparent in case this prognosis is based on the assessment of the actual mutational events. The specific features of the mutagenesis of various genomic elements, whose mechanisms, initiation rate, and biological consequences significantly differ from each other, usually are not discussed.

Meanwhile, without changing our views regarding the mechanism of the effect of ionizing radiation on the genetic material of biological objects, it is impossible to expect that the contradictions between the experimental data and predictions based on simplified conceptions and models of the mutagenic action of ionizing radiation on living objects will be resolved.

**Heterogeneity of target sensitivity in living objects. Molecular, cellular, organ, species diversity of the damaging effects of ionizing radiation**

One of the reasons behind the existing difficulties is the fact that mutational events also occur at a relatively high frequency due to endogenous events. Thus, the frequency of spontaneous mutations in the structural genes in human cells is 5 ・10^-11^ per base per round of cell division [15]. The frequency of nucleotide substitutions in genes leading to substitutions in the amino acid sequences in the corresponding protein products is discussed in this case. The diploid genome of higher mammals contains approximately 6・10^9^ nucleotides, and roughly 10% of those are found in the coding regions; i.e., this frequency is typical of 6 ・10^8^ genomic nucleotides. Hence, approximately 1・10^-3^ mutations emerge in the coding regions of the structural genes in each genome in each round of replication.

Today the frequency of mutations is determined per nucleotide in the human haploid set (3 ・10^9^ nucleotides) using a comparative analysis of the completely sequenced genomes of parents and two monozygotic twins. The frequency of spontaneous substitutions estimated using this method reaches 1.1 ・10^–8^ per nucleotide per round of replication [16].

Every second 107 cells in a normal human organism undergo division [17]; hence, every second 103 new cells containing nucleotide substitutes exclusively in the protein-coding regions are formed. It is important to point out that actual mutations are discussed in this review.

However, these mutations are preceded by DNA lesions at the potential mutation sites, since they can either be easily repaired or become actual mutations. They usually emerge due to hydrolysis, oxidation, or electrophilic influence on DNA molecules. These reactions occur as a result of an exogenous impact, including the impact of ionizing radiation; they can also result from endogenous metabolic processes. The endogenous events cause a large number of various DNA lesions [17]. Thus, for instance, apurine/apyrimidine (AP) sites in DNA may occur as a result of spontaneous hydrolysis or DNA-glycosylase-aided excision repair. AP sites are quickly repaired by AP endonuclease, which catalyses the hydrolysis of the 5’-phosphodiester bond with subsequent removal of 3’-phosphate (aided by the lyase activity of DNA polymerase β) [18]. Nakamura and Swenberg [19] determined the number of AP sites in DNA from tissues. They found out that the number of such sites in the genomes of cells isolated from most human and rodent tissues reaches 50,000–200,000 (i.e. ~ 10^-5^ –10^-4^ per nucleotide). It is clear that such lesions occur 6–7 orders of magnitude more frequently as compared to the nucleotide substitutions in the structural genes. The typical AP sites induce nucleotide substitutions (typically А→Т) or may result in frameshift mutations. These mutations have been found in the microsatellite loci of a plasmid treated with Н_2_О_2_. The number of AP sites increases when the cells are treated with oxidizing or methylating agents.

It has been known for a relatively long time that the action of oxygen radicals leads to the emergence of a large number of oxidized bases in DNA, as well as to DNA breaks. Base modifications that occur due to the other oxidizing processes are discussed less frequently. Thus, polyunsaturated fatty acids, one of the main components of membrane phospholipids, are characterized by high sensitivity to oxidation and are the main target of oxygen radicals [18]. During the oxidation of polyunsaturated fatty acids, bifunctional electrophilic groups capable of interacting with DNA bases are synthesized, giving rise to exocyclic compounds. These modified bases carrying the exocyclic groups disrupt the double-stranded DNA and are considered to be potentially highly mutagenic.

Exocyclic ethylene groups (denoted by ε: εА, εСand N^2^,3-εG) have been identified in the DNA isolated from various human tissues; their level increases with an increase in the concentration of oxygen radicals. N^2^,3-εG can be found in rodent tissues subjected to oxidizing stress. The amounts of εА, εС are increased in patients with Wilson’s disease and the diseases associated with accumulation of copper and iron in the liver [20]. The rate of oxidation of unsaturated fatty acids increases during the accumulation of metal ions. The level of ethylene groups is also increased in DNA isolated from the polyps of patients suffering from familial adenomatous polyposis, and, interestingly, in the DNA of leucocytes isolated from the blood of women whose food contains large amounts of unsaturated fatty acids; this effect is absent in males [20].

The amounts of εА, εС increase during the promotion stage of a tumor in a two-stage mouse skin carcinogenesis model [21]. The treatment of a tumor promoter using phorbol ester (tetradecanoylforbol-13-acetate) has resulted in a 9- to12-fold increase in εА and εС, respectively. The increase in the amount of DNA lesions correlates with the induction of fatty acid oxygenase (8-lipoxygenase).

Special attention has recently been brought to bear on the C → T transitions in CpG-islets, since these mutations are commonly found in organisms affected by cancer and in a number of other pathologies. Cytosine methylation is considered to be an important factor in the occurrence of such transitions.

The rearrangements in large DNA segments may underlie the occurrence of chromosomal translocations, resulting in particular in a loss of heterozygosity, which is very commonly observed in tumor cells. The chromosomal alterations begin with breaks in both DNA strands, which are induced by oxidative stress or enzymatic cleavage during chromatin reorganization (e.g., DNA topoisomerase II). Approximately 10 doublestrand DNA breaks per cell cycle in the form of blocked replication forks occur during DNA replication [22]. It is evident that the factors giving rise to double-strand breaks and the methods to repair them significantly contribute to the processes of endogenous mutagenesis.

Studies of animals with various knock-outs of the genes encoding the enzymes used to repair doublestrand breaks have demonstrated the significance of these modifications for the occurrence of mutational events at the nucleotide and chromosome levels.

It is obvious that the high frequency of spontaneous mutational events leads to the fact that mutagenic effects are attributed not to the occurrence of the lesions but to the activity of their repair (e.g., those in radiation-resistant species known as *Deinococcus radiodurans,* which are capable of withstanding 5000 Gy [23]).

The investigation into the mechanisms of spontaneous lesions in the genetic material (each of which can be converted into a mutation with an unpredictable effect for the cell, its clonal progeny, and the multicellular organism in general) indicates that these events are multistage by their nature. Supplementing this cascade with ionizing radiation will be an additional factor increasing the probability of conversion of potential DNA lesions into mutations.

The next level of control of the genetic stability in a multicellular organism, which prevents the accumulation of mutant cell clones, is based on the various options of cell death available to genetically deficient cells, as well as the participation of immune system cells in the elimination of mutant clones [24]. It is obvious that during this stage the ionizing radiation can have a dual effect: it can increase the portion of dying mutant cells and reduce the rate at which they are eliminated by suppressing the effector cells of the immune system.

The reproduction of mutant cell clones of the germinative line and the emergence of mutant progeny are controlled by a cascade of events, each of which can be modified by ionizing radiation.

It is interesting to point out that embryonic preimplantation mortality is increased and embryonic fission is slowed down *in vitro *in СС57W/Mv mice under the influence of absorbed ionizing radiation at a dose of 0.4–0.5 Gy (with respect to the control) as was confirmed in our experiments [25]. It should be emphasized that the preimplantation embryonic losses in mammals are difficult to control, and they manifest themselves through a decrease in the fertility of the population.

The analysis of the events of mutagenesis at the gene, cell and organism levels demonstrate that the characteristics of its alterations under the influence of low-dose ionizing radiation depend on many factors, which are connected both with genetically determined processes and with the modifying effect of the environmental factors. The complexity of the interactions between the genetic and environmental components prevents the examination of individual characteristics of mutagenesis as an integral indicator of the effect of low doses of ionizing radiation on multicellular organisms. A decrease in population fertility seems to be the most objective indicator.

**Conceptions of the radiation doses capable of causing damage to living objects**

Significant amounts of experimental observations of the consequences of increasing the level of ionizing radiation in various species, including the human one, have been accumulated. The most commonly used methods for assessing the health consequences of increased levels of ionizing radiation are as follows: determining the growth in the incidence of oncological diseases; the frequency of occurrence of dividing cells (generally in the peripheral blood) containing lesions in the genetic apparatus; and the proportion of children born with congenital anomalies [26]. Today an increase in the incidence of thyroid cancer remains the only indicator of worsening of population health as a result of the Chernobyl accident that generates no disputes [26]. The features of the data regarding the cytogenetic anomalies in somatic cells include high individual variability of the characteristics of chromosomal apparatus destabilization and a lack of clearly defined linear relationships between the degree of karyotype destabilization and radionuclide contamination of the habitat [27] and the amount of cesium isotopes in an organism [28, 29].

Laboratory and field studies have demonstrated that an increase in the level of ionizing radiation in a number of cases (even in the low-dose range, up to 20–30 mGy) is accompanied by an increase in the frequency of individual species characterized by an increased proportion of somatic cells with various mutations [27, 29]. Meanwhile, after the bombing of the cities of Hiroshima and Nagasaki, it is generally considered that an increase in the frequency of cancer due to the increased level of ionizing radiation can be definitively traced only if the absorbed dose exceeds 100 mSv/year. The data accumulated following the Chernobyl accident indicate that even significantly lower doses can be damaging; this fact requires the theory of damaging doses to be reconsidered [30]. The explicit contradiction between the observation of the effects at the levels of cell populations and individual species and the statistical analyses of average populations can be due to a number of reasons. It is of significant importance to elucidate these reasons in order to develop objective techniques to predict the damaging effects of low-dose ionizing radiation and to search for integral indicators of this effect.

Leukemia was the first oncological disease whose incidence was connected to the consequences of the atomic bombings in Hiroshima and Nagasaki. The maximum incidence was observed 5 years after the bombings. Ten years later, an increase in the growth of solid tumors was recorded. Chronic lymphocytic leukemia, pancreatic and prostate cancer, and endometrial cancer were the most frequently identified types. Even 55 years after the atomic bombings, 40% of the people initially included in the Atomic-bomb Survivor Research Program are still alive; this fact makes it possible to assess the longterm consequences of the exposure. It was determined that the relative risk of developing various conditions per dose unit is higher among survivors of the bombings than among those who were subjected to medical radiation exposure. The risk of developing a disease among employees of atomic stations and miners is on average comparable to that observed among some population groups which survived the atomic bombings. The risk of somatic conditions among those who were subjected to primary exposure of identical doses decreases with increasing age at which the exposure occurred. The frequency of various conditions of respiratory, gastrointestinal, and vascular systems is increased among survivors of the atomic bombings [31, 32].

The connection between the doses absorbed by the embryos and the incidence of leukemia in children was analyzed in England, Scotland, Greece, Germany, and Belarus following the Chernobyl accident. The total doses absorbed by the embryos varied from 0.02 mSv in England to 0.06 mSv in Germany, 0.2 mSv in Greece and 2 mSv in Belorus. A statistically significant increase in the risk of developing leukemia in newborns was identified at the peak of the exposure, between July 1, 1986, and December 31, 1987, as compared with children born between January 1, 1980, and December 31, 1985, and between January 01, 1988, and December 31, 1990. In certain countries, the risk increases nonmonotonously with respect to the dose absorbed: it increases abruptly at low doses, followed by a decrease at high doses. The findings have been discussed in connection with the mechanisms of embryo/cell death at high doses and dose-dependent induction of DNA repair. The accumulated results show the need for reconsidering our views on the absorbed doses that could be harmful to embryos [33]. In children who received high doses of ionizing radiation in the period between 0 and 5 years after birth, an increase in leukemia incidence was also observed, which correlated with the absorbed doses (over 10 mGy) [34]. Nonetheless it is important to emphasize that the relationship between cancer development and various factors exerting influence simultaneously (especially for leukemia), the difficulties of diagnosing and classification, as well as treatment success, hinder the assessment of the contribution of radionuclide contamination following the Chernobyl accident to the dynamics of oncopathology and early mortality in European countries [35].

Meanwhile, there are data available that indicate that the incidence of congenital developmental anomalies among children born to fathers who participated in the cleanup effort after the Chernobyl accident exceeds the average frequency recorded in the Russian Federation [36]. An increase in the incidence of congenital developmental anomalies in children born in the regions with high levels of radionuclide contamination following the Chernobyl accident was revealed by the analysis of the Belarus National registry of congenital anomalies for the period of 1983–1999 performed by G.I. Lazyuk *et al. *[37].

It was demonstrated that high and low doses of ionizing radiation have a nonthreshold effect on the cardiovascular system. At least two mechanisms are involved in the formation of these effects: the impact on the formation of macrophage-enriched atherosclerotic plaques due to inflammation processes on the vessel wall and a decrease in the cardiac muscle blood supply as a result. The manifestation of the pathology of the cardiovascular system following the irradiation exhibits a large lag-phase, especially after exposure to a low dose [38].

It was discovered that the number of boys born in Bavaria and Denmark in 1987 after the Chernobyl Nuclear Power Plant accident was higher than that of girls [39]. The mortality rate among newborns increased considerably in 1987–1988 [40].

Young people who received doses of ionizing radiation *in utero* had considerably lower IQ scores as compared to those in the control group of the same age. The differences were limited to the verbal IQ score; the nonverbal IQ score was not affected. These effects were not identified in the group of people who were exposed to radiation after 16 weeks of prenatal development [41].

The incidence of cytogenetic anomalies in cells isolated from the peripheral blood and bone marrow at various laboratory and wild-type small murine rodents that reproduced in the alienation zone of the Chernobyl Nuclear Power Plant (CN PP) was determined. This zone is a unique system model to study the population genetic transformations caused by a change in the direction and intensity of natural selection. An abrupt change in the entire complex of ecological factors occurred on a limited territory following the accident. Representatives of various taxonomic groups, including higher mammals, dwell successfully in spite of these changes. It is important to emphasize that the main culprit in the ecological catastrophe (the emission of radionuclides) has been well established. It should be highlighted that it is impossible to simulate the action of interdependent and interrelated changes in various conditions of population reproduction under the influence even of a single environmental stress factor in a laboratory (e.g., an increase in radionuclide contamination), whereas it is typically impossible to analyze the inheritance of induced changes in generations of families under field conditions.

**Table 1:** The frequency of micronuclei occurrence per 1,000 erythrocytes (EMN) isolated from the peripheral blood of СС57W/Mv mice of various ages from the Chernobyl and Kiev populations during different seasons

Season	EMN, control, ‰		EMN, Chernobyl, ‰	
	2.5–3.5 months	14–16 months	2.5–3.5 months	14–16 months
Winter 1993	2.5 ± 0.7	2.7 ± 0.9	10.0 ± 1.0	4.0 ± 1.0
Summer 1994	3.0 ± 0.8	3.2 ± 0.7	6.5 ± 1.0	3.8 ± 0.3

In order to evaluate the possible direction of population genetic changes under the influence of an increase in ionizing radiation in the alienation zone of the CN PP, with allowance for the genetic heterogeneity of bioindicator species in field conditions, a comparative analysis of long-term changes in generations of genetically homogenous laboratory СС57W/Mv mice and in species of field voles captured between 1994 and 2001 in the alienation zone of the CN PP in locations with various levels of radionuclide contamination was carried out in the present study. The cytogenetic variability in generations of the genetically homogenous population was analyzed using two populations of СС57W/Mv mice bred at the Institute of Molecular Biology and Genetics of the National Academy of Sciences of Ukraine: the Chernobyl group (a specialized vivarium located in a 10-km radius from the CN PP) and the control group (a vivarium in Kiev). These populations were kindly provided by S.S. Malyuta, an academician of the National Academy of Sciences of Ukraine. The bone marrow cells isolated from mice of first and second generations of the Kiev population (K-1, K-2) and the first, second, fifth, seventh, and tenth generations of the Chernobyl population (Ch-1, Ch-2, Ch-5, Ch-7 and Ch-10) reproducing in the specialized vivarium at a rated dose of absorbed radiation of approximately 0.6 Gy per animal were analyzed.

The mutational spectra were determined in representatives of the field vole species (*Microtus arvalis* and *Clethrionomys glareolus)* captured in the alienation zone of the CN PP at locations with low levels of radionuclide contamination (< 5 Cu/km^2^), which were considered spontaneous (conditional control) under conditions of an intermediate level of radionuclide contamination (~200 Cu/km^2^ – Yanov, rated dose of absorbed radiation of approximately 0.6–0.8 Gy/year) and with high levels (500–1000 Cu/km^2^, Glubokoe lake, Chistogalovka, “Red Forest,” rated dose of absorbed radiation of approximately 0.9–1.1 Gy/year).

The following cytogenetic characteristics were taken into consideration in the animal bone marrow cells: two types of aneuploidy, polyploidy, the frequency of occurrence of metaphases with chromosomal aberrations (CA), centric fusions known as Robertsonian translocations (RB) and asynchronous separation of chromosomal centromeric regions at the end of the metaphase (ASCR ). The percentage of aneuploid cells was determined in two different variants: the cells with a loss or gain of the chromosome number, more than one (general aneuploidy, A1) and aneuploid cells with a number of chromosomes 2*n*± 1 (A2). The numbers of binuclear leukocytes (BL) and leukocytes with micronuclei (LM) were determined in cells with an intact cytoplasm using the same preparations. The mitotic index (MI) and the frequency of occurrence of BL and LM were determined per 1,000 cells.

A significant increase in the frequency of cytogenetic anomalies was identified in the mutational spectra of the Chernobyl population of СС57W/Mv mice (in particular, the metaphases with chromosomal aberrations: 0.9 ± 0.2% in the control group and 6.0 ± 2.0% in the test group). Meanwhile, the responses to identical levels of increased ionizing radiation were different to the statistically significant level in groups of linear mice of various ages (*Table 1, 2*) and in generations of the experimental population (*Table 3*).

The data in *Table 1* show that the frequencies of occurrence of erythrocytes with micronuclei in the con trol groups of “young” and “old” animals determined in different seasons show no statistically significant differences. However, this figure was significantly higher in the Chernobyl “young” animals than that in the control population and in the “old” animals. The results of these experiments (*Table 1*) gave grounds to assume that the age-related changes are accompanied by a decrease in erythroblast sensitivity to damaging effects. It can be also expected that in this case the processes of physiological adaptation in “old” animals as compared to the ”young” group are connected to the long-lasting action of chronic low-dose exposure to radiation. It was determined that this physiological adaptation was accompanied by an increase in the number of dividing cells and a certain acceleration of the cell cycle, if the determination is based on the number of binuclear leukocytes per round of mitosis in bone marrow cells isolated from the groups of the “old” control and the Chernobyl animals (*Table 2*).

**Table 2:** The frequencies of cell deletions and cytogenetic anomalies in ”young” and ”old” CC57W/Mv mice from the control (Kiev) and the Chernobyl groups

Mice group	Age, months	MI, ‰	BL, ‰	LM, ‰
Mice from the ”Young” control group (Kiev)	2-3	6.8 ± 0.5***	4.5 ± 0.7	5.2 ± 0.3**
Mice from the ”Young” experimental group (Chernobyl)	2-3	5.6 ± 0.7	9.0 ± 1.4*	14.4 ± 2.4**
Mice from the “Old” control group (Kiev)	12-18	3.5 ± 0.6***	7.1 ± 1.3	10.5 ± 1.3*
Mice from the “Old” test group (Chernobyl)	12-18	7.0 + 1.0*	5.0 + 0.8*	6.0 + 0.8*

*P < 0.05

**P < 0.01

***P < 0.001

Note. Here and in Tables 3 and 4, MI is the number of metaphases per 1,000 cells; BL is the number of binuclear leucocytes per 1,000 cells; LM is the number of mononuclear leucocytes with micronuclei per 1,000 cells.

**Table 3:** The variability of cytogenetic characteristics in CC57W/Mv mice in generations (Ch-1, Ch-2, Ch-5, Ch-7, Ch-10) reproducing under conditions of the specialized vivarium of the CNPP as compared with the control populations of K-1 and K-2 (average values)

Population	Aneuploid cells, %		Polyploid cells, %	The frequency of metaphase occurrence,%			Number per 1000 cells, ‰		
	A1	A2	PC	RB	CA	ASCR	MI	BL	LM
K-1	23 ± 1	10 ± 1	8 ± 1	10 ± 1	0.9 ± 0.4	0.7 ± 0.3	6.0 ± 0.7	6.0 ± 0.6	6.0 ± 0.7
K-2	27 ± 5	10 ± 1	4 ± 1	9 ± 3	2.0 ± 0.1	2.0 ± 1.0	7.0 ± 0.6	5.0 ± 0.5	5.0 ± 0.4
Ch-1	30 ± 2	12 ± 1	4 ± 2	11 ± 3	6.0 ± 2.0	5.0 ± 1.0	7.0 ± 1.0	10.0 ± 1.5	14.0 ± 2.0
Ch-2	28 ± 3	5 ± 3	9 ± 2	8 ± 2	6.0 ± 3.0	7.0 ± 1.0	7.0 ± 1.0	5.0 ± 0.9	6.0 ± 1.0
Ch-5	34 ± 5	5 ± 1	4 ± 1	3 ± 1	0.5 ± 0.2	1.0 ± 1.0	8.0 ± 1.0	7.0 ± 0.4	6.0 ± 0.4
Ch-7	35 ± 2	7 ± 1	2 ± 1	8 ± 1	4.0 ± 1.0	4.0 ± 1.0	7.0 ± 0.7	8.0 ± 1.0	5.0 ± 2.0
Ch-10	20 ± 2	3 ± 1	3 ± 1	1.0 ± 0.2	4.5 ± 1.0	2.5 ± 0.4	6.0 ± 0.7	7.0 ± 1.0	5.0 ± 0.4

Note. Here and in Table 4, A1 is general aneuploidy, A2 is aneuploidy 2n± 1 chromosome; PC is the fraction of polyploid cells; RB is the fraction of cells with centric fusion of chromosomes (Robertsonian translocations); CA is the frequency of occurrence of metaphases with chromosomal aberrations; ASCR is the proportion of cells with asynchronous separation of the centromeric regions of the chromosomes at the end of the metaphase.

The analysis of the incidence of cytogenetic anomalies in the bone marrow cells of the “young” СС57W/ Mv mice in the sequential generations (Ch-1, Ch-2, Ch-5, Ch-7, Ch-10) reproducing under conditions of chronic exposure to increased levels of ionizing radiation allowed to identify a nonlinearity of the changes in a number of cytogenetic characteristics in generations of genetically homogenous animals (*Table 3*). It turned out that the incidence of such cytogenetic characteristics as RB, CA, and ASCR , which are directly connected with intra-chromosomal aberrations, decreases in the 5^th^ generation, increases in the 7^th^ generation, and decreases again in the 10th generation. A similar pattern of variability in the generations was identified for a fraction of the second type of aneuploid cells (2*n *= 40 ± 1 chromosome). The incidence of other types of cytogenetic anomalies was higher only in the first generation of Chernobyl mice as compared to that in the control populations (*Table 3*).

**Table 4:** The incidence of various cytogenetic anomalies in the bone marrow cells of bank vole species captured at locations characterized by different levels of radionuclide contamination

The frequency of occurrence of metaphases, %						The number per 1,000 bone marrow cells, ‰		
A1	A2	RB	PP	ASCR	CA	MI	BL	LM
Control								
33.7 ± 6	9.0 ± 3.5	14.0 ± 3.5	0.5 ± 0.5	6.2 ± 3.6	1.2 ± 0.7	3.2 ± 0.6	3.5 ± 0.6	5.5 ± 1.5
Total: Janov for the period between 1997 and 1999								
31.2 ± 2 4	8.9 ± 3.7	13.9 ± 6.0	6.9 ± 5.6	10.1 ± 4.1	8.1 ± 4.0	5.7 ± 1.0	5.2 ± 0.8	3.2 ± 0.8
“Red Forest” 1999								
34.6 ± 6.2	10.5 ± 3.0	22.6 ± 3.6	1.2 ± 0.7	9.6 ± 1.3	3.5 ± 0.8	5.2 ± 1.2	3.7 ± 1.1	6.5 ± 0.7
“Red Forest” 2001								
35.2 ± 2.8	6.3 ± 1.1	12.9 ± 3.1	0.5 ± 0.4	11.8 ± 2.8	0.9 ± 0.3	8.0 ± 2.5	9.8 ± 1.7	8.0 ± 1.2

**Table 5:** The incidence of various cytogenetic anomalies in the bone marrow cells of common vole species captured at locations characterized by different levels of radionuclide contamination

The frequency of occurrence of metaphases, %						Per 1,000 mononuclear lymphocytes, ‰		
A1	A2	PC	RB	SA	ASCR	MI	BL	LM
Control								
44.4 ± 5.1	8.6 ± 0.8	0.9 ± 0.5	1.0 ± 0.5	2.5 ± 0.6	16.5 ± 4.9	4.5 ± 0.9	5.0 ± 0.8	3.0 ± 0.4
2001, Chistogalovka, Glubokoe lake, ~500 Cu/km^2^								
26.5 + 2.7	3.1 + 0.8	1.8 + 0.4	0.3 + 0.3	2.5 + 0.3	17.6 + 4.1	6.1 + 0.6	7.8 + 1.6	3.1 + 0.5

The nonlinear variability in generations of genetically homogenous mice, which was determined from the frequency of occurrence of cells with intrachromosomal deficiencies, may be an indication that the intensity of the aberrations caused by a prolonged action of ionizing radiation in a low-dose range is comparable to the activity of damage repair processes, elimination of damaged cells, and the rate of division of undamaged substituting cell clones. A multitude of factors controlling the mechanisms of physiological adaptation to ionizing radiation in animals, the comparability of the intensity of the action of multidirectional factors may result in a sequential increase and decrease in the incidence of the cytogenetic anomalies that occur in the presence of a relatively constant level of a damaging agent in genetically homogenous animals.

An analysis of genetically heterogeneous populations of field voles captured in various years at locations with various levels of radionuclide contamination allowed to obtain data supporting the fact that the selection for radioresistant animals became clearly evident in 1999 and 2001 among populations of bank voles and common vole which inhabit regions characterized by high levels of radionuclide contamination (*Tables 4 and 5*).

Thus, the bank vole populations captured in 1999 and 2001 at locations with the highest level of radionuclide contamination mainly contained animals whose bone marrow cells were characterized by an incidence of cytogenetic anomalies that not only was higher than that in the conditional control population, but was sometimes lower with respect to certain characteristics (*Table 4*). It was determined that an increase in the number of radioresistant species among the bank voles was most prominently evident in the “Red Forest” population (1000 Cu/km^2^). This selection was not observed at the locations characterized by a significantly lower level of radionuclide contamination (Janov, ~200 Cu/km^2^).

Similar data were obtained when studying common vole populations (*Table 5*).

Thus, the accumulation of hypothetically radioresistant species in generations of genetically homogenous laboratory mice and a decrease in the incidence of certain types of cytogenetic anomalies at a lower rate than that normally observed in the control populations were revealed in the genetically heterogeneous populations of two types of field vole species captured at locations characterized by high levels of radionuclide contamination.

It seems that the sources of these nonlinear responses to ionizing radiation in the low-dose range can be numerous, can occur at different phases of the ionizing radiation exposure, and at different levels of organization of multicellular organisms. Thus, a complex cascade of biochemical events can be initiated due to the induction of free-radical processes in cells by ionizing radiation [42]. The accumulation of free radicals results in the activation of antioxidant enzymes that limit the free radical processes (they include superoxide dismutase, catalase, glutathione peroxidase, and glutathione reductase [43]). The activation of these enzymes can be tissue- and organelle-specific. For instance, the change in the antioxidant activity in mitochondria was found to be directly connected with the stability of the chromosomal cellular apparatus. Characteristic “radial” markers (Robertsonian translocations, dicentrics, and circular chromosomes) occur in the bone marrow cells of mice lacking functional mitochondrial superoxide dismutase [44]. An increase in the radioresistance of human chromosomes with an increase in the telomerase activity [45] and its decrease due to damage to chromatin proteins, which prevents the occurrence of double-strand breaks in DNA during mutation, have been described [46]. The existence of a “substrate” induction of DNA repair processes was demonstrated: it was discovered that a large number of double-strand breaks in DNA occurring following the irradiation of human fibroblasts at a dose of 2 Gy are repaired much more rapidly as compared to the numerous breaks that occur after irradiation at a dose of 200 mGy [47]. It can also be expected that some contribution to the nonlinear responses of multicellular organisms to the same dose of ionizing radiation can also be made by intracellular interactions, such as changes in the ratio between highly specialized to poorly differentiated cell populations. The ratio between the effector cells of the immune system, which differ from each other in terms of their radiosensitivity, as well as the presence of the antigen structures recognized by killer cells on the surface of the plasma membrane of the damaged cells, can contribute to this response in mammals [48].

Thus, the findings suggest that the incidence of certain types of cytogenic anomalies in generations of genetically homogenous and genetically heterogeneous populations of small murine rodents undergo nonlinear changes under prolonged exposure to low doses of ionizing radiation. It can be surmised that this nonlinearity is due to the multiplicity and multidirectionality of the radiation-induced repair processes existing at the cellular and subcellular levels, as well as to the action of the exogenous factor that is comparable to the former in terms of the intensity of the damaging effect. The nonlinearity of the effects hypothetically disappears only when the intensity of the damaging action of ionizing radiation significantly exceeds the capabilities of the multifactor mechanisms of adaptation.

It was discovered in our experiments using three different strains of laboratory mice (the Chernobyl population – mice from a specialized vivarium in the 10-km zone of influence of the CN PP; the control group – mice from the vivarium in Kiev) that each strain under control conditions has its own spectrum of spontaneous mutations in bone marrow cells, and that only certain characteristics of this spectrum varied with animal age and the season during which the research was carried out [49]. Thus, an increase in aneuploidy (chromosome loss) with an increase in age and during the transition to the summer season was typical of the C57BL/6 mice. In the CC 57W/Mv mice, the age and the seasonal changes were mainly associated with intrachromosomal defects (chromosomal aberrations), and in BALB/c mice they were associated with the percentage of polyploid cells. Under conditions of an increased level of ionizing radiation in the specialized vivarium near the CN PP (absorbed doses of approximately 0.5–0.6 Gy/year), an increased incidence was observed only for those anomalies that were characterized by spontaneous instability under control conditions. For instance, an increased frequency of the occurrence of aneuploid cells was observed in the C57BL/6 strain, and an increased frequency of metaphases with chromosomal aberrations was observed in CC 57W/Mv mice. Thus, an increase in the doses of ionizing radiation in this case did not result in the development of new characteristics in the mutational spectra of mice but caused an increase in the spontaneous instability of the individual strain-specific characteristics of these spectra.

The same tendency was revealed for the voles captured in the areas with elevated levels of radionuclide contamination. Only cytogenetic abnormalities, whose increased variability is species-specific for voles dwelling in unpolluted areas, accumulate in bone marrow cells: metaphases with Robertsonian interchromosomal fusions for the bank voles and aneuploidy for the common voles. The data obtained suggest that elevated levels of ionizing radiation (within the investigated range) increase the incidence of cytogenetic anomalies in laboratory strains of mice and in voles, which are characterized by high strain- and species-specific variability (in mice) under control conditions. The analysis of these mutational spectra is further complicated by the fact that individual chromosomes of the experimental animals exhibit a pronounced predisposition to a particular type of cytogenetic abnormalities.

A comparative analysis of the incidence of various chromosomal breaks in blood cells extracted from 14- to15-year-old children was carried out. One group consisted of children who received ionizing radiation at a dose of approximately 30 mSv during their prenatal development; the second group consisted of children who received approximately the same dose, but in the course of their entire lives as they lived in the contaminated areas (approximately 1.5 mSv/year) [50, 51]. The frequencies of occurrence of cells with cytogenetic abnormalities were found to be almost identical in these two groups of children; however, the first group (acute exposure during the prenatal period) was characterized by a statistically significant increase in the number of cells with stable chromosomal abnormalities, such as translocations, inversions, and insertions. These data indicate that cell clones with the aforementioned anomalies are accumulated in the blood of children. Since a certain parallelism between the frequency of mutational events in populations of somatic and generative cells has been identified along with the fact that these types of cytogenetic abnormalities can significantly complicate the progression of meiosis it can be expected that the children who were exposed to ionizing radiation in the prenatal period of their lives will face reproductive problems [50, 51].

It is interesting to mention that the tendency towards increasing sensitivity to ionizing radiation with an increase in the complexity of an organism has been established a long time ago; i.e., the more ancient the species the more resistant it is to ionizing radiation. The mean half-lethal dose of radiation is approximately 4–6 Gy for mammals; it reaches 30 Gy for colibacillus (*Escherichia coli)*. The absolute record holder in this respect is the bacterium *D. radiodurans*, whose individual cells survive and retain their reproductive capacity after being exposed to 5000 Gy [23]. It was discovered that immediately after irradiation with a dose of 3000 Gy, nearly all the genomic DNA of this organism decomposes into small fragments; a single doublestrand break was on average induced per 27-kb-long DNA segment, and 3 h after the exposure the genome initiates the recovery process without any significant accumulation of mutations in the structural genes. A species-specific ability to restore the genomic integrity rather than a unique stability of the genetic material to ionizing radiation is observed in this case. It was found that the ability of this species to repair DNA damage is associated with drought-resistance genes, the mutations in which lead to the disappearance of this unique radiation stability of the species.

It is believed that the improvement in the accuracy (“resolution”) of the genetic methods for assessing chromosomal aberrations at low-dose exposures will ensure a more accurate determination of the relationship between low-dose radiations and induced mutational events. This assumption was supported by the data pertaining to the occurrence of new mutations in highly polymorphic DNA sequences in children born to irradiated parents [52, 53]. However, the study by Veynber *et al.* [52], in which RAPD-PCR (Random Amplification of Polymorphic DNA) markers but not ISSR-PCR (Inter Simple Sequence Repeats) markers were used, provided data on the occurrence of new mutations in children born to those who participated in the cleanup effort after Chernobyl. Dubrova *et al.* [53] revealed an increased frequency of mutations in three of the eight investigated minisatellite loci in children whose parents lived in areas with high radionuclide contamination. In other words, the results of the analysis of the mutational events induced by low-dose exposure were dependent on the type of DNA markers used (RAPD-PCR , but not ISSR-PCR ) and the investigated loci. Hence it follows that no unambiguous data on the genetic effects of low-dose ionizing radiation, which would be independent of the analysis technique and the variability features of an individual loci, can be obtained using the available methods for detecting mutational events directly in the DNA.

Attempts to assess the genetic consequences of the action of ecotoxic factors have been made for an appreciably long period; two main trends can be distinguished in this development. One consists in searching for the molecular genetic systems associated with detoxification and antioxidant enzymes; the second one is the population genetic investigation of the dynamics of allelic variants in the genetic systems associated with resistance to these factors.

The biomarkers of xenobiotic metabolism are usually subdivided into genes whose products are involved in the metabolic activation of promutagens (procarcinogens) with the occurrence of short-lived, highly toxic derivatives (in particular, cytochrome P450 genes) and genes whose products control their detoxification (e.g., glutathione-S-transferase and N-acetyltransferase). The direct link between the genetically determined polymorphism of these enzymes and the induction of cytogenetic abnormalities by a number of xenobiotics, reproductive function disorders, and the development of certain types of tumors in humans has recently been discovered using PCR [54–56].

The second area is being investigated less successfully. It is based on research into the changes in the structure of the populations that are subjected to environmental stress. The distribution of allelic variants and the genotypes of the structural genes and anonymous (in terms of their function), highly polymorphic DNA, whose involvement in the formation of sensitivity to genotoxic agents has not been established, are being investigated.

Associations have been discovered between human resistance to ionizing radiation and the occurrence of certain genotypes, mainly with respect to the transferrin and haptoglobin loci [57, 58]. Japanese researchers have also described an increased frequency of certain genotypes with respect to the MHC genes in “Hibakusha” long-livers (survivors of the atomic bombings of the cities of Nagasaki and Hiroshima), which is presumably associated with a particular state of the immune system. The latter contributes to an increased resistance to a number of common diseases [59].

Success in searching for the biomarkers of resistance to genotoxic effects largely depends on the quality and adequacy of the models used to study the population genetic consequences of various types of environmental stress.

It is obvious that the high frequency of spontaneous mutational events leads to the fact that mutagenic effects are attributed not to the occurrence of the lesions but to the activity of their repair (e.g., those in radiation-resistant species known as Deinococcus radiodurans, which are capable of withstanding 5000 Gy [23]).

The identification of the genes and gene ensembles whose inheritance is preferable and is associated with the selection for resistance to new environmental conditions may result in the development of the so-called individual “genetic passports” of resistance to physical and chemical environmental pollution. These data enable to control the population genetic structure of the species and facilitate its transformation in the desired direction.

The basic data obtained during our investigations were thoroughly described in the monograph [49] and performed on different types of mammals using genetic biochemical, molecular genetics, and cytogenetic methods.

A shift in the spectra of organ-specific isoenzymes occurs in a number of farm animal species under the influence of elevated levels of ionizing radiation (an absorbed dose of 0.6–0.8 Gy/year), mostly in kidney and heart tissues. The heart tissue starts to express a number of isoenzymes, which are typically found in the poorer specialized muscle tissue. No significant increase in the number of individuals with constitutive mutations has been identified during population-based studies of various mammal species. The following observations have been made with respect to generations of cattle that received absorbed doses of approximately 0.8 Gy/year (^137^Cs): a) reduced fertility and increased mortality in newborn calves; b) disruption of the equiprobable inheritance of individual allelic variants – elimination of some and preferable inheritance of the other allelic variants; c) displacement of the genetic structure of the parent generation typical of dairy cattle towards less specialized forms; d) changes in the genetic structure that coincided with the population genetic effects of such biotic and abiotic stress factors as the selection for resistance to bovine leukemia infection and introduction into new reproduction conditions. Therefore, the findings suggest that the main response to a prolonged exposure to low-dose ionizing radiation consists not in the induction of the emergence of new genes but in the preferential selection of new gene combinations in the generations. Hence, this concept is consistent with the principles of the evolution theory elaborated by I.I. Schmalhausen [60]: a variation of the selection criteria leads to the preferential reproduction of the least specialized species, as observed in generations of cattle under the influence of various environmental stress factors.

Conventional biochemical and molecular genetic markers were used to investigate the features of the genetic structure of animal groups, which allowed to analyze a number of polymorphisms in a number of loci encoding plasma proteins. Furthermore, markers of the intron sequence of the leptin gene and the exon 4 of the k-casein gene and DNA fragments flanked by microsatellite loci (ISSR-PCR markers) were also employed. The informative nature of utilizing additional characteristics of the genetic structure of the species was also assessed: an analysis of the patterns of interloci gene associations was carried out [61]. The investigated loci were parts of various linkage groups [62]. Two groups of syntenic genes (the transferrin and ceruloplasmin genes – chromosome 1; vitamin D receptor, k-casein, and hemoglobin – chromosome 6) and four non-syntenic genes (amylase 1 gene – chromosome 3, leptin – chromosome 4, purine nucleoside phosphorylase – chromosome 10, and post-transferrin 2 – chromosome 19) were investigated.

The following data were obtained during the investigation of the genetic structure of a number of generations of cattle bred under conditions of high radionuclide contamination in the alienation zone of the CN PP. Only one animal with a mutation in the locus encoding transferrin was identified in the second generation. Disruption of the equiprobable transfer of the allelic variants from parent to offspring was detected in certain loci; changes in the results of the assessment of disequilibrium with respect to the linkage in a number of loci were also recorded. It was demonstrated that, regardless of consanguineous mating under conditions of increased radionuclide contamination, the heterozygosity in the generations did not decrease.

Therefore, the occurrence of population genetic changes in animals under conditions of environmental stress was initially established using this model, and these changes were shown to be directed towards the preferential reproduction of heterozygotes with respect to the structural genes and “anonymous” DNA segments (ISSR-PCR markers).

Next, we compared the population genetic consequences of elevated levels of ionizing radiation and the influence of the other biotic and abiotic environmental stress factors on the genetic structure, which was evaluated using a variety of molecular genetic markers, inbreeding groups of different breeds of cattle. The following mechanisms of the influence of environmental factors were considered. The analysis of the Red Steppe breed included two groups of animals that differed in terms of their resistance to the influence of a biotic stress factor (infected and uninfected with the bovine leukemia virus) – from farms in the Kherson region, which was relatively unaffected by technogeneous pollution; and from farms in Kirovograd and Donetsk, which were characterized by elevated levels of chemical contamination (an abiotic factor). Three groups of animals belonging to the Pinzgau breed were examined in connection with their reproduction in the plains, mountains, and under high-altitude conditions (an abiotic factor). The gray Ukrainian breed was represented by two groups of animals: those from the Kherson region (the original habitat) and from the Altai region and Siberia (new conditions – an abiotic factor). In the Holstein breed, the effect of the abiotic factor was determined by comparing the genetic structure of two groups, one of which reproduced at farms in the relatively uncontaminated Kherson region and the other (experimental herd) reproduced on the “Novoshepelichi” farm located in the alienation zone of the CN PP (radionuclide contamination being approximately 200 Cu/km^2^, an abiotic factor). The population genetic studies were conducted using a variety of markers, including electrophoretic variants of proteins, restriction site polymorphism, and ISSR-PCR markers.

It was determined that the influence of environmental stress factors can result in a significant genetic differentiation in animal groups, which in some cases was higher than the interbreed differences. Two genes encoding the vitamin D receptor and apurine-nucleoside phosphorylase have been identified. Obvious differences in the frequencies of the alleles of the latter genes have been found in groups of cattle of the same breed living under various environmental stress conditions (chemical pollution, introduction into new reproduction conditions, and infection with the bovine leukemia virus). This suggests the existence of universal characteristics of the population genetic response of cattle to the influence of various environmental stress factors [63].

An analysis of the reproductive characteristics of experimental herds of cattle can reveal the mechanisms behind the changes in the genetic structure which occurr in the descendants of parents exposed to ecotoxic factors and deletions of a number of alleles and genotypes associated with increased sensitivity. Thus, the experimental herd exposed to high levels of ionizing radiation (absorbed dose of 0.8–1.1 Gy/year) included the parental generation (F0) consisting of three cows (Alpha, Beta, Gamma) and a bull named Uranus captured in 1987 near the CN PP and of 13 cows imported between 1990 and 1993 to the “Novoshepelichi” farm (Pripyat) from relatively uncontaminated areas. The F0 generation of cows (a total of 16) from the experimental herd born in the uncontaminated area gave birth to a total of 96 calves (0.93 ± 0.03 calves per cow per year), 20 of which (21%) did not survive 3 months after birth. The first generation (F1) born on the experimental “Novoshepelichi” farm was significantly different from the parent generation with respect to this figure. Thus, 21 cows of the 36 cows from the F1 generation were sterile (58%), and only 15 of them yielded offspring (F2 generation, 0.73 ± 0.06); 13 calves died before reaching three months of age (26%). Four cows from the F2 generation gave birth only to 10 calves (F3) in 2–4 years, i.e. 0.94 ± 0.06 calves per cow per year. It should be mentioned that most of the calves from the F1 generation, which did not survive, were bulls (six heifers and 14 calves); the sex ratio amongst 13 dead calves from the F2 generation was approximately the same (seven heifers and six bulls).

Therefore, the changes in the genetic structure of the offspring received from parents exposed to the influence of ecotoxic factors can be attributed to a decrease in fertility and an increase in infant mortality (carriers of the allelic variants associated with increased sensitivity to a given factor).

## CONSEQUENCES OF LIVING IN RADIOACTIVE AREAS ON HUMAN HEALTH

There are many “radioactive” areas located in different parts of the earth; various population genetic characteristics of the populations inhabiting them have been identified in some of them. Thus, extensive studies of the populations living in areas characterized by a highly radioactive background were conducted (Kerala state in India, Guangdong province in China), where the exposure of the population to radiation ranges from 0.6 to 10 cGy per year. No increase in the number of human congenital diseases was revealed during these investigations [64, 65]. The screening of over 40,000 pregnant women living in areas with a high radioactive background in Brazil demonstrated no increase in the incidence of spontaneous abortions and congenital anomalies, although the incidence of chromosomal aberrations in blood cells isolated from the local population was slightly higher than that in the control areas [66].

Ramsar County in Iran is best known for its annual absorbed dose of 260 mSv; the global average dose is equal to 3.5 mSv/year. The residents of Ramsar County are not characterized by elevated rates of mortality or birth of children with congenital developmental abnormalities.

Meanwhile, distinctive differences in the radioresistance of blood cells isolated from the local population living in this area are observed, in contrast to residents of areas characterized by a low natural radioactive background. Thus, the exposure of the cell cultures of peripheral blood isolated from the inhabitants of Ramsar County to a dose of 1.5 Gy resulted in a significantly smaller increase in the number of cells with cytogenetic anomalies as compared to the blood cells of the control group [67].

The published results of studies of populations living in “radioactive” areas suggest that the selection process for increased radioresistance occurs in these locations from generation to generation. Thus, 125,079 residents of the “radioactive” province were examined in China during the period between 1979 and 1995; 10,415 deaths and 1,003 cancer cases have been analyzed. It was found that the cancer mortality rate in the “radioactive” province was lower than that among residents of the control area [68]. In another study, the authors concluded that a 3- to 5-fold increase in the level of ionizing radiation did not increase the risk of oncological pathologies [69].

No significant differences in the incidence of congenital defects have been identified among newborns (26,151) from the “radioactive” province in India (approximately 35.0 mSv/year) and among newborns (10,654) in the control group [70]. Screening of the inhabitants of another “radioactive” province (over 70 mSv/year) in India (a total of 400,000 people, 100,000 of those lived in the “radioactive” part of the province) showed no differences in the incidence of oncological pathologies due to a high level of external γ-radiation [71].

Annual detection of oncological conditions per 100,000 people in the populations inhabiting Indian regions, which vary from each other by only 0.03 mSv/ year with respect to the external radiation exposure proportionately decreases from one area to another one simultaneously with an increase in the background level of ionizing radiation by 0.03 mSv/year – from the hypothetical incidence of oncological conditions being equal to 79 : 100,000 people, under conditions of “zero” level of external exposure. The authors arrived at conclusion that an increase in ionizing radiation decreases the risk of developing cancer [72].

It should be emphasized that among the 116,000 people evacuated from the Chernobyl zone, only approximately 5% of them received a dose of ionizing radiation exceeding 100 mSv/year, and this very dose (almost 3 times lower than that in Ramsar County) is considered to be the limit exceeding which leads to an increase in the incidence of oncological conditions [73].

Thus, the actual danger is not the received dose of ionizing radiation but its “novelty” to this particular population, species, or species community. It is obvious that an annual increase in the absorbed dose by 3.5mSv will not result in any health consequences among the residents of Ramsar Country, but for most European populations whose previous generations have not been exposed to doses exceeding 1 mSv/year such an increase can lead to the elimination of radiosensitive species from the gene pool, thus resulting in a change in the genetic structure of the populations.

Hence, there is a wide range of doses of ionizing radiation within the natural conditions, which are compatible with the ability of various organisms (including humans) to survive and reproduce. This complicates the evaluation of the biological (including genotoxic) effects of low-dose ionizing radiation.

## POPULATION GENETIC CONSEQUENCES OF CHRONIC LOW-DOSE EXPOSURE TO IONIZING RADIATION IN VARIOUS SPECIES OF MAMMALS: LABORATORY MICE STRAINS, FIELD VOLES, CATTLE

Our own experimental data suggest that a chronic exposure to elevated levels of ionizing radiation causes no increase in the number of mutant individuals among the investigated species. An increased frequency of somatic cells with cytogenetic anomalies was not accompanied by qualitative changes in comparison to the spontaneous mutational spectra, since the increase was only observed for the indicators of the instability of the chromosomal apparatus that possessed genotypic features in the strains of mice and field vole species.

A comparative analysis of the mutational spectra in field voles conducted in different years showed that the number of individuals of different species with a high frequency of mutant cells in the bone marrow decreased gradually over time despite the persistence of high levels of radioactive contamination at the capture locations. The frequency of occurrence of individual species with high levels of cytogenetic anomalies among the common voles and bank voles was significantly higher in 1996 than that in the relatively ”uncontaminated areas, and in animals captured at the same locations but during the later years, in 1999 and 2001. Thus, metaphases with chromosomal aberrations in the common voles were observed in the control group at a frequency of 2.5 ± 1.5%, and in Chistogalovka at a frequency of 3.6 ± 0.8%, 5.0 ± 2.3%, and 2.5 ± 0.3% in 1996, 1999, and 2001, respectively. These metaphases were encountered in the bank vole species of the control group at a frequency of 1.2 ± 0.7%; in the «Red Forest » in 1996, 1999, and 2001 at a frequency of 7.3 ± 3.4%, 3.5 ± 0.8%, and 0.9 ± 0.3%, respectively.

It is important to emphasize that this decrease indicating the gradual accumulation of radioresistant species among the bank vole species was only observed in the animals captured in the “Red Forest” area, which was characterized by a very high level of radionuclide contamination (> 1000 Cu/km^2^), in contrast to the animals inhabiting the areas with lower levels of radionuclide contamination (Janov, ~200 Cu/km^2^). Thus, the rate of selection for radioresistance is higher when the level of radionuclide contamination is higher. It is noteworthy that even in the areas of the alienation zone characterized by a high level of radionuclide contamination (i.e. “Red Forest”), an accumulation of radioresistant species was detected only in 1999; i.e., 13 years after the Chernobyl accident, after 26 generations of voles (voles breed twice a year).

It is interesting to mention that similar data on the selection of the individuals resistant to adverse environmental conditions were obtained by us for various agricultural species reproducing under conditions of the biosphere reserve (Lake Khovsgol, Mongolia) and in the area of risk-associated livestock farming in the southern part of the Gobi Desert. [74] A comparative analysis of the frequency of occurrence of erythrocytes with micronuclei in the blood samples of local Mongolian cattle, sheep, and yaks reproducing under various ecological and geographical conditions was performed: northwestern Mongolia, Khovsgol region, biosphere reserve; southern Mongolia, the area adjacent to the Gobi Desert – a zone of risk-associated livestock farming. The number of erythrocytes containing micronuclei were determined in a smear of 3,000 cells and the value was expressed in parts per million (‰). The frequencies of occurrence of erythrocytes containing micronuclei were similar across various species reproducing under the same environmental conditions, but significantly differed in animals from different ecological and geographical regions. Thus, the frequencies of occurrence of red blood cells containing micronuclei were significantly higher in the area of the biosphere reserve under favorable conditions of reproduction than those in animals of the same species in the risk-associated livestock farming zone. The frequency of occurrence of erythrocytes containing micronuclei among sheep in the Khovsgol area (22) was found to be 5.3 ± 0.4 ‰; in cattle (7) – 4.6 ± 0.7 ‰; in yaks (7) – 3.2 ± 0.6 ‰; in sheep in the Gobi Desert (10) – 0.9 ± 0.1 ‰; in cattle (7) – 1.8 ± 0.6 ‰; and in yaks (7) – 0.3 ± 0.2 ‰. These findings suggest that the long-term influence of highintensity environmental stress factors contributes to the selection of animals (over a number of generations) with increased tolerance of the genetic apparatus to unfavorable environmental conditions.

The disruption of equiprobable transmission of allelic variants of several molecular genetic markers and an increase in heterozygosity were observed among generations of the experimental “Novoshepelichi” herd of the black and white Holstein cattle. The frequency of occurrence of leukocytes containing micronuclei in the parental generation was higher at a statistically significant level (P < 0.05) as compared to these values in the first, second and third generations of animals born in the zone with an elevated radionuclide contamination. However, this parameter was significantly lower in the third generation (P < 0.01) as compared to that in the second generation. The frequency of occurrence of binucleated leukocytes in the peripheral blood smears was also significantly higher in the parental generation than those in the first and second generations of animals. Thus, the radioresistance of animals born under conditions of the elevated levels of ionizing radiation increases over generations as evidenced by the incidence of cytogenetic abnormalities in the peripheral blood smears. No carriers of Robertsonian translocations were identified, which are often found in uncontaminated areas among representatives of species with acrocentric autosomes.

A comparative analysis with respect to a complex of molecular genetic markers in the experimental herd of black and white Holstein cattle from the relatively uncontaminated breeding areas, as well as the representatives of the ancient primitive breed known as the Ukrainian gray breed allowed to observe a convergence of the genetic structure of animals from the experimental herd born under conditions of elevated levels of radionuclide exposure with the gene pool of this ancient breed in contrast to the parental group of individuals. This shift in the genetic structure of the gene pool of the experimental “Novoshepelichi” herd originally belonging to a specialized dairy breed towards a more primitive species was observed with respect to the allelic variants of the structural genes and DNA fragments flanked by inverted microsatellite repeats. It can be expected that these changes are a universal population-genetic response of cattle to the influence of various environmental stress factors.

## CONCLUSIONS

The following data are common for our own findings and to the published data. The main problem that populations of different species (including humans inhabiting the areas contaminated with radionuclides after the Chernobyl accident) face is not the absolute value of the dose of ionizing radiation, but the novelty of these doses to the populations. The main genetic effects for populations of various species consist not in the increase in the number of mutant organisms but in the fact that some of the genes are excluded from their reproduction as a result of selection against radiosensitive organisms. In other words, new genes do not emerge, but the ”old” ones associated with high sensitivity of the organisms to new conditions of reproduction abandon the population. There is some indirect evidence suggesting that the less specialized organisms of the species turn out to be more adapted to new conditions.

This ”reversion” to the more primitive, developmentally and evolutionarily earlier forms of life is observed at various levels of organization of biological material: a shift in the organ-specific isozymatic spectra to developmentally earlier versions; displacement of the population-genetic structure in generations to the predominance of less specialized forms; *in utero *exposure resulted in a decrease exclusively in the verbal IQ score among young people [41], which is a more recent evolutionary acquisition of humans in comparison to the non-verbal IQ score, which is based on older structures. The actual genetic consequences of the Chernobyl disaster for human populations will not be completely elucidated soon, since children born after the year 1986 have only recently entered the reproductive period of their lives.

It is interesting to note that data have been obtained indicating that the first-priority destruction of the youngest biological systems in evolutionary terms is a relatively universal biological rule [75].

The accumulated data allow one to formulate four major laws of the Chernobyl disaster. We believe they can be universally applied to the consequences of all fundamental environmental changes associated with natural and man-made disasters and crises. These laws are as follows: 1) not everyone who was supposed to be born is being born after the Chernobyl accident; 2) the selection against specialized forms of life and preferential reproduction of less specialized forms characterized by higher resistance to adverse environmental factors occurs; 3) the response to the same dose of ionizing radiation depends on its “novelty” for the population of preceding selection for resistance to such doses in the ancestral generations; and 4) the actual consequences of the Chernobyl accident for human populations will be available for analysis no earlier than 20 years from now, since the generation subjected to direct damaging influence has only recently entered its reproductive period. It should be emphasized that the increase in the incidence of even the thyroid pathology resulting from the Chernobyl accident reduces the chances of reproductive success in its carriers.
